# The comparison of muscle strength and short-term endurance in the different periods of type 2 diabetes

**DOI:** 10.1186/2251-6581-13-22

**Published:** 2014-01-29

**Authors:** Boshra Hatef, Farid Bahrpeyma, Mohammad R Mohajeri Tehrani

**Affiliations:** 1 Department of physical therapy, Faculty of Medical Science, Tarbiat Modares University, Tehran, Iran; 2 Endocrinology and Metabolism Research Center, Tehran University of Medical Sciences, Tehran, Iran

**Keywords:** Type 2 diabetes mellitus, Isokinetic, Strength, short-term endurance, Knee extensor, Knee flexor

## Abstract

**Background:**

Patients with type 2 diabetes (T2DM) are subjected to reduction in the quality and oxidative capacity of muscles. The effect of duration of diabetes on the muscle endurance response is not clear and strength as well.

**Objective:**

The aim of this study was the assessment of strength and endurance of knee extensor and flexor in the patients with T2DM < 10 and T2DM > 10 years in comparison with age, sex, BMI, ABI and PAI-matched health control subjects.

**Methods:**

Isometric maximal peak torque (MPT) of knee extensor and flexor before and after 40 isokinetic repetitions with velocity of 150 degree/s were recorded in 18 patients with T2DM < 10 Y , 12 patients with T2DM > 10 Y and 20 matched health control (HC) groups.

**Results:**

Both diabetic patient groups had significant lower isometric and isotonic knee extensor and flexor strength than HC. The endurance indices indicated that whereas the isometric MPT of flexor movement was reduced after isokinetic protocol in the both patient groups in comparison with HC, the less decline was seen in the isotonic torque and work during isokinetic protocol in the T2DM > 10 Y group in comparison with two other groups. The HbA1c and FPG were significantly correlated with strength not with endurance indices.

**Conclusions:**

It seems the progression of diabetes accompanied with vascular, neural and muscular deficits activate, some adaptive and compensatory processes which can maintain muscle performance.

## Background

Insulin resistance, metabolic inflexibility, muscular and neuromuscular impairments develop the decrease of muscle strength and quality especially in the lower limb. Strength reduction has been associated with increase of neuropathy, HbA1c and duration of diabetes [1-9]. However, there are not enough evidences about fatigability of diabetic muscles [1,5,6,10]. In the study of Andersen et al., 44 patients with long-term T2DM more than 20 years were compared with 44 matched health participants. The results showed that healthy participants were fatigued more than long-term T2DM during 30 maximal isokinetic movements of knee and ankle at 180 degree/s was not correlated neither to the severity of neuropathy nor to the metabolic control (blood glucose and HbA1c) for any of the muscle groups [1]. In contrast, two different isokinetic studies, 20 repetitions with 120 degree/s and 30 repetitions with 180 degree/s, revealed that patients with T2DM had lower resistance to fatigue in the knee flexor and extensor in comparison with matched health subjects respectively [5,6]. The distribution and density of muscle fibers has important role in the fatigue muscle [11]. In the T2DM patients not only sarcopenia and diminished muscle quality were observed [7,12] but also the proportion of fatigue-resistant type 1 muscle fiber was decreased and the proportion of the least fatigue-resistant type 2b muscle fibers was increased [13-15]. The changes in muscle fiber distribution are related closely to progress of diabetes. it is suggested that the increase in the number of type 2b fibers may be induced by the decrease in the number of capillaries around muscle fiber [13], number of mitochondria and oxidative capacity [16], GLUT4 and the insensitivity to insulin [17]. It is expected in parallel to the change of muscle type over time, the endurance response should be changed [11]. But there is no evidence to investigate the effect of diabetes with different duration on the endurance property of diabetic muscles. Practically, because the exercise has important role in the control and treatment of diabetes, the identification of muscle strength and endurance properties can be helpful to design the better exercise protocol [18]. The assessment of strength and endurance in the static and dynamic conditions in the short intensive exercise in the patients with the less and more than 10 years of diabetes in the comparison with sex, age, BMI, PAI (physical activity index) and ABI (ankle brachial index) matched healthy subjects may clarify the relation of strength and endurance changes with the duration of diabetes.

## Methods

### Subjects

T2DM patients voluntary were recruited by medical doctors from internal medicine and endocrine clinics of Tehran. The inclusion criteria were: age between 25-70 years, without any severe or uncontrolled cardiac disease diagnosed by specialist, no intermittent claudication and foot ulcers, no muscular disorders or rheumatoid arthritis and 1.5 > ABI > 0.9. Finally thirty patients participated in the study and they categorized into two groups according to the duration of diagnosed diabetes, 18 patients with less than 10 years of disease in the T2DM < 10 Y group and 12 patients with equal and more than 10 years of disease were placed in theT2DM > 10 Y group. The healthy control (HC) group consisted of twenty individuals who were matched in terms of sex, age, BMI, ABI and PAI with two other groups. All inclusion criteria and also HbA1c < 6%. Were considered for HC group written informed consent was obtained from all participants and the protocol was approved by the medical ethical committee of the Tarbiat Modares University.

### Procedure

HbA1c and FPG by blood sample and ABI by Doppler ultrasound in the supine position from dominant leg were measured in all participants. The ABI is calculated by dividing the systolic blood pressure at the ankle by the systolic blood pressure in the ipsilateral arm the those who were in normal range between 0.91-1.3 entered the study [19]. The level of physical activity had been measured by scoring of PAI [20]. For each person, the total score of multiplying of exercise intensity and duration and frequency was calculated and it matched with activity category. All participants had been distributed in the sedentary, poor and fair categories.

The test for all participants was performed in the afternoon. The patients were asked to take prescribed medications. The brachial pressure and finger blood glucose were measured before test.

### Isometric and isokinetic muscle testing

Isometric maximal peak torque [Newton meter] (MPT) was assessed for the knee flexion and extension of the dominant leg which determined by the preferred leg for kicking a ball with an isokinetic dynamometer (HUMAC NORM, USA). Before the study, all subjects received instructions about the procedure and performed a warm-up session (quadriceps and hamstring stretching 30 second two times and 5 minutes free load ergonomic cycling and five trial submaximal repetitions isotonic and two isometric). After 2 minutes resting, test was started. To determine maximal voluntary isometric torque, participants were positioned in a 85 degree of hip and 75 degree of knee flexion (the optimal length for maximal knee extensor performance [21]), straps were placed around the pelvis and trunk. The subject’s knee joint center was aligned with the rotation center of the dynamometer. The lower leg was strapped to the dynamometer lever arm resistance by using a calf pad, 5 cm proximal to the lateral malleolus. The settings of the chair and lever arm for each subject were adjusted by calibrations of the instrument. The Verbal encouragement was given during the test and asked participants to press maximally lever arm to extension for three times, each press was kept for 3 s and a rest period of 30 seconds was given between consecutive contractions. They repeated this protocol for knee flexion as well. Isokinetic protocol (40 isoconcentric knee extension/flexion performed at 150 degree/s through 0-75 knee flexion) was followed after a few seconds and the subjects were instructed to push and pull "as hard and fast as possible" through the full available ROM at every repetitions without holding breath and resting. The heart rate was controlled during the protocol with finger heart beat controller device attached to the middle finger of the opposite hand. The isometric protocol as explained before were repeated after 2 minutes rest.

### Data analysis

#### *Muscle strength variables*

The highest torque output from the three MVCs was selected as isometric MPT [21]. The highest torque from 40 repetitions was selected as Isotonic MPT. All joint moments were normalized by body mass [6]. The ratio of extensor MPT to flexor was also considered for both isometric and isotonic records.

#### *The endurance variables*

The fatigue index (FI) was calculated as the ratio of the mean work of the last 5 repetitions to the mean work of the highest, five consecutive repetitions within the first fifteen repetitions. The peak torque (PT) of 40 repetitions were normalized to isotonic MPT and the slope of line across the relative PT of 40 repetitions was indicated the amount of force decline during isokinetic protocol. Isometric MPTs were repeated after isokinetic protocol, then the different of isometric MPT of extensor, flexor and ratio of extensor on flexor from those before isokinetic protocol were also measured. The Table 1 represents all dependent strength and endurance variables of knee extensor and flexor torques (Table 1).

**Table 1:** Sorting and identification of dependent variables

**Strength indices**	WEXT1 (Nm/Kg)	MAX torque of EXT isometric/weight
	WFLEX1 (Nm/Kg)	MAX torque of FLEX isometric/weight
	WEXT1/WFLEX1	
	WTEXT (Nm/Kg)	MAX torque of EXT isotonic/weight
	WTFLEX (Nm/Kg)	MAX torque of FLEX isotonic/weight
	WTEXT/WTFLEX	
**Endurance indices**	EXTBA (Nm/Kg)	WEXT2- WEXT1
	FLEXBA (Nm/Kg)	WFLEX2- WFLEX1
	RatioBA	WEXT2/WFLEX2 -WEXT1/WFLEX1
	Slope EXT	The slope of line across the relative isotonic EXT torque of 40 repetitions
	Slope FLEX	The slope of line across the relative isotonic FLEX torque of 40 repetitions
	EXT fatigue index (%)	Mean 5 last works/mean 5 highest works from 15 first works of EXT
	FLEX fatigue index (%)	Mean 5 last works/mean 5 highest works from 15 first works of FLEX

*WEXT1 and WFLEX1 were MAX isometric torque before 40 isotonic repetitions, WEXT2 and WFLEX2 were MAX isometric torque after 40 isotonic repetitions.

#### *Statistical analysis*

Chi-squared was used to assess differences of distribution of sex and PAI and ANOVA was performed to determine differences of age, weight, length, BMI, ABI, HbAlc, FPG and glucose test between three groups (HC, T2DM < 10 Y and T2DM > 10 Y). ANCOVA was carried out to assess the effect of grouping and sex on the strength indices in which age was considered as covariate expected the ratio. Endurance indices were analyzed by two-ways ANOVA in which grouping and sex were main factors. Pearson correlation was performed to detect correlation between variables including HbAlc, FPG and glucose test and strength and endurance indices. A p-value of <0.05 was considered as significant.

## Results

We included 18 participated patients with T2DM < 10 years, 12 patients withT2DM > 10 years and 20 HC subjects. Demographic and blood parameters are presented in Table 2. In general, diabetic subjects with good to moderate blood glucose control included in this study. The HC group was 10 years younger than T2DM > 10 Y group (P = 0.02). HbAlc, FPG and glucose test of the HC group was lower than two diabetic groups (P < 0.000) while two diabetic groups were not different significantly.

**Table 2:** Demographic and blood characters

	**HC**	**T2DM < 10 Y**	**T2DM > 10 Y**	**P value**
Number of cases (women/men)	20 (10/10)	18 (9/9)	12 (6/6)	1
Age (years)	49.55 ± 10	52.11 ± 9.2	59.17 ± 7.1	0.02*
Weight (Kg)	73.68 ± 7.7	77.61 ± 12.5	77.72 ± 12.4	0.47
Height (cm)	167.89 ± 9.2	164.33 ± 8.3	166.09 ± 11	0.51
BMI	26.25 ± 3	28.71 ± 4.1	28.54 ± 3.6	0.09
Duration of diabetes (years)		4.8 ± 2	15.5 ± 7	
Medication (insulin + drugs/drugs) (N of cases)		1/17	3/9	
PAI (sedentary/poor/fair) (%)	63.2, 26.3, 10.5	66.7, 27.8, 5.6	45.5, 54.5,0	0.83
ABI	1.17 ± 0.08	1.22 ± 0.09	1.14 ± 0.14	0.43
HbA1c (%)	4.7 ± 0.8	7.02 ± 1.5	7.3 ± 1.4	0.000
FPG (mmol/l)	91 ± 12.6	141.65 ± 36.9	160.91 ± 31.4	0.000
Blood glucose (mmol/l)	114.05 ± 17.7	167.39 ± 44.23	205.20 ± 84.24	0.000

Data are mean ± SD. *Post Hoc between health and T2DM > 10 Y groups, **Post Hoc between healthy and T2DM > 10 and T2DM <10 Y groups.

### Muscle strength

**Figure 1:**
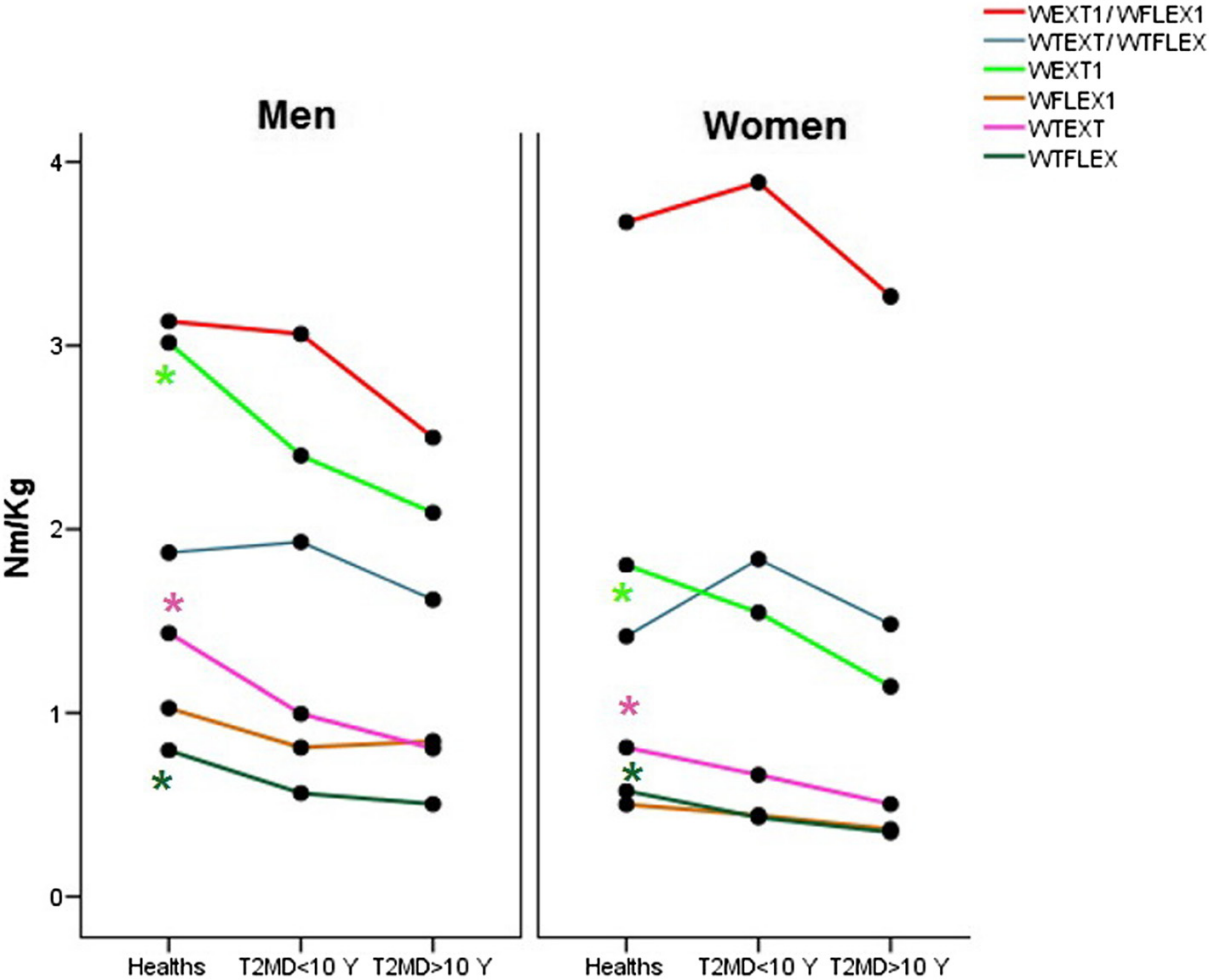
**Line plots of strength indices in the three groups (HC, T2DM < 10 Y and T2DM > 10 Y) separately in the men and women.** Significance is indicated as*.

No interaction effect was found between grouping and sex in the all variables. The HC group had higher isotonic MPT in the EXT and FLEX movements than two diabetic groups while isometric MPT only in the EXT movement was higher in the HC group than two diabetic groups. Although T2DM > 10 Y group had lower MPT than T2DM < 10 Y group but the difference was not significant. All MPT were higher in the men than women (Table 3). The ratio of MPT EXT on FLEX in both isometric and isotonic conditions were not been affected by grouping and the isometric ratio of extensor on flexor was only affected by sex (Figure 1).

**Table 3:** F and P value of ANCOVA test in the strength indices main effect of grouping (HC, T2DM < 10 Y and T2DM > 10 Y) and sex (men, women)

	**WEXT1**	**WFLEX1**	**WTEXT**	**WTFLEX**
Main effect of grouping (HC (n = 20), T2DM < 10 Y (n = 18), T2DM > 10 Y (n = 12))	3.99, .026**	2.15, .12	10.57, .000*	8.53, .001*
Main effect of sex (Men (n = 25), Women (n = 25))	38.54, .000	69.66, .000	39.04, .000	12.22, .001

Data are F, Significance of two way ANCOVA. **Post Hoc between HC and T2MD > 10 Y groups, *Post Hoc between HC and T2MD < 10 and >10 Y groups.

### Muscle endurance

**Figure 2:**
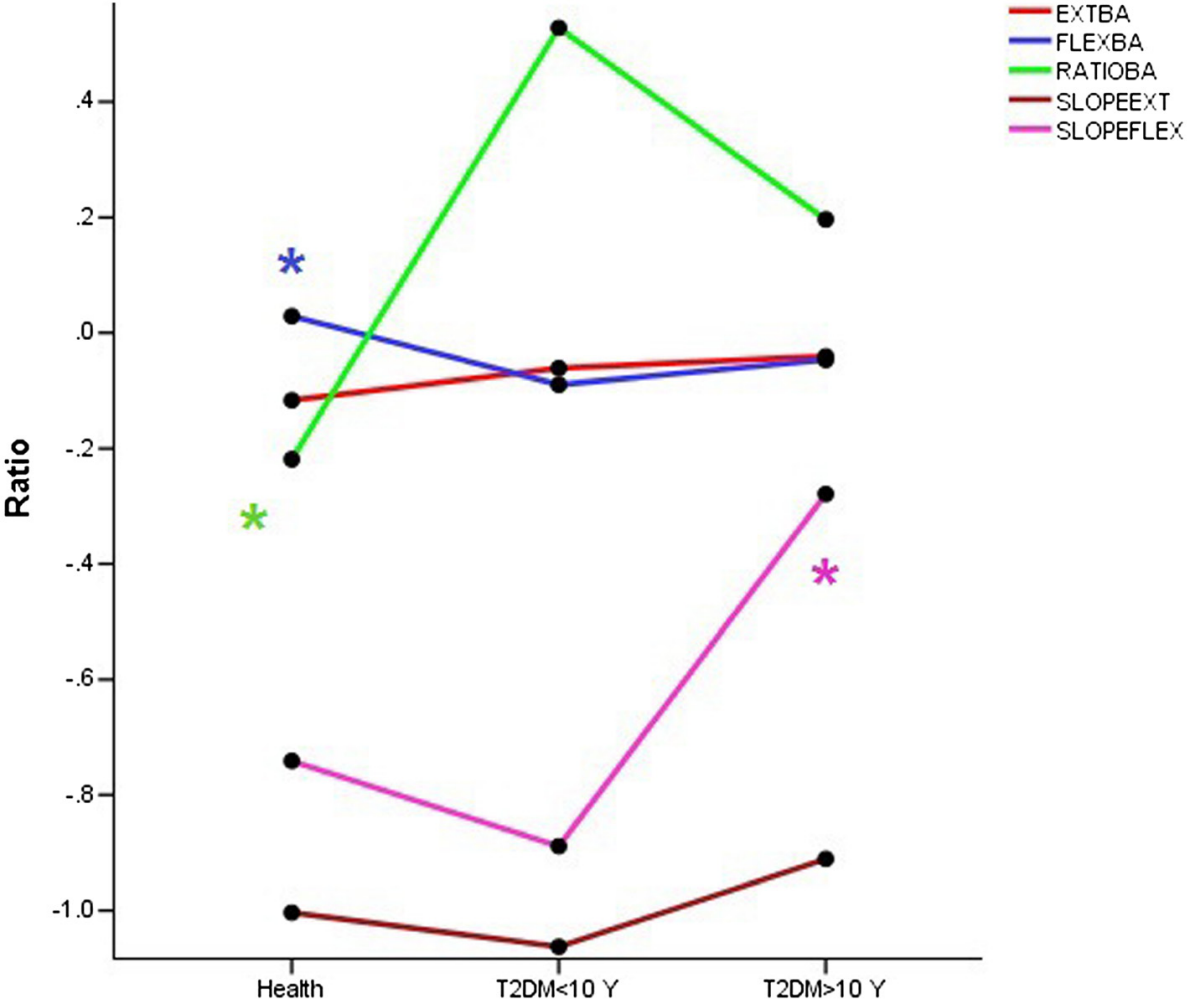
Line plots are indicated endurance indices in the three groups (HC, T2DM < 10 Y and T2DM > 10 Y).

No interaction effect was found between grouping and sex in all variables. The Table 4 showed the main effect of grouping and sex on the endurance indices. The endurance indices of extension movement demonstrated that no significant different presented between three groups. Nevertheless The means T2DM > 10 Y group were more close to means of the HC group in comparison with the T2DM < 10 Y group (Figure 2). The effect of sex showed the men had higher EXTFI than women (P = 0.02). It means the men were more resistant to fatigue in the extensor muscles than women.

**Table 4:** F and P value of two-way ANOVA of endurance indices, main effect of grouping (HC, T2DM < 10 Y and T2DM > 10 Y) and sex (men, women)

	**Fatigue index**		**Slope**		**Isometric after-before**		**Ratio EXT/FLEX after-before**
	**EXT**	**FLEX**	**EXT**	**FLEX**	**EXTBA**	**FLEXBA**	**RatioBA**
Effect of grouping	.67, .51	4.05, .050**	.30, .73	7.97, .001*	.50, .60	11.17, .0001¥	8.18, .001§
(HC (n = 20), T2DM < 10 Y (n = 18), T2DM > 10 Y (n = 12))							
Effect of sex	5.60, .02	1.54, .22	1.47, .23	.005, .94	.39, .53	.33, .56	.02, .88
(Men (n = 25), Women (n = 25))							

Data are F, Significance of two way ANOVA. **Post Hoc between T2MD < 10 Y and T2MD > 10 Y, *Post Hoc between T2MD > 10 Y and HC and T2MD < 10 Y groups, ¥Post Hoc between HC and two T2MD groups, §: Post Hoc between HC and T2MD < 10 Y groups.

**Figure 3:**
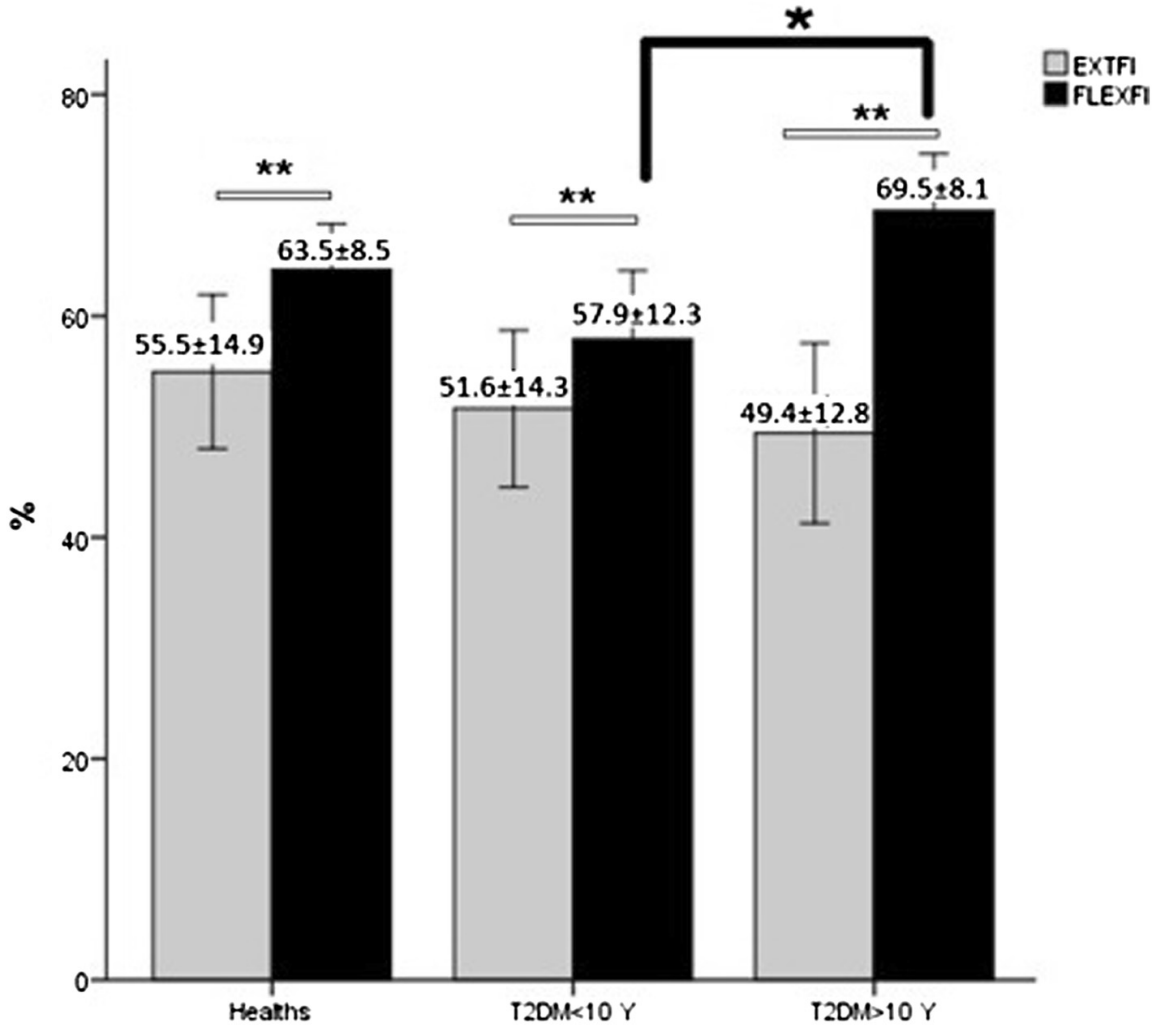
**Bar plots are indicated mean and SE of EXT and FLEX FI in the three groups (HC, T2DM < 10 Y and T2DM > 10 Y).** ** is indicated significance between EXT and FLEX FI, * is indicated significance between T2DM < 10 Y and T2DM > 10 Y groups.

In the flexor movement, the results showed no significant effect of gender on endurance indices. The HC group could kept the isometric MPT of FLEX after isokinetic protocol more than both diabetic groups while the mean of T2DM > 10 Y group was more close to HC group. The difference between after and before of isometric MPT ratio showed that the T2DM < 10 Y group significantly more than HC group. Flexion IF and slope of isokinetic protocol showed that the patients of T2DM > 10 Y group were more resistant to fatigue than T2DM < 10 Y group even than HC group that was significant only in the FLEX slope. In the three groups the EXT FI significantly was lower than FLEX FI (P < 0.000) (Table 4 and Figure 3).

### Correlations

HbA1c, FPG and glucose test were correlated negatively with isotonic MPT of both movement and isometric MPT of extension movement while only FPG had positive correlation with EXTBA and RATIOBA and negative correlation with FLEXBA from the endurance indices. In the patients the years of diabetes had significant negative correlation with isotonic MPT of both movement and positive correlation with slope flex (Table 5).

**Table 5:** the correlation of the bloods with muscle factors

	**WEXT1**	**WFLEX1**	**WTEXT**	**WTFLEX**	**EXTBA**	**FLEXBA**	**RATIOBA**	**Slope flex**
**FPG**	-.37 (.023)	-.10 (.56)	-.44 (.007)	-.41 (.013)	.38 (.02)	-.43 (.009)	.41 (.013)	.17 (.29)
**HBA1C**	-.45 (.002)	-.17 (.25)	-.54 (.000)	-.47 (.001)	.26 (.08)	-.27 (.07)	.26 (.07)	.14 (.34)
**Glucose test**	-.25 (.08)	-.16 (.27)	-.37 (.008)	-.35 (.01)	.19 (.19)	-.11 (.44)	.17 (.22)	.30 (.03)
**Years of diabetes**	-.34 (.06)	-.14 (.43)	-.44 (.01)	-.38 (.03)	.25 (.17)	.27 (.12)	-.09 (.62)	.61 (.000)

Data are Pearson Correlation (P value), n = 50.

## Discussion

### Muscle strength

The hypothesis of the study that the diabetic patients had weaker muscles than healthy subjects was confirmed. But there was no significant difference between patients of T2MD > 10 Y and T2MD < 10 group. It may be because two patient groups were matched in the PAI, ABI, age, BMI and sex or limited number of subjects. The ratio of EXT MPT on the FLEX MPT in the isometric or isotonic conditions were not affected by diabetes. Most of strength indices had significant negative correlation with HbA1c and FPG and glucose test and years of diabetes. The effect of sex clearly indicated that the men were significantly stronger than women and there was no interaction effect of grouping and sex.

The effect of diabetes on the muscle strength specially in the lower limb strongly has been confirmed in many studies also correlation of the decrease of strength with the intensity of neuropathy, HbA1c, FPG and duration of diabetes has been determined [2-7,9,22,23]. The muscle strength is affected by age, sex and BMI and [24,25]. However, if the muscle strength is normalized to cross sectional area or muscle mass detected by DEXA, the gender role would be reduced [7,26]. Therefore, the study of Ijzerman et al. that demonstrated lower isometric maximal torque of knee extension and flexion normalized on the weight in the T2DM with and without neuropathy than healthy subjects was not enough reliable because the distribution of gender was significantly different between groups [6].

the effect of diabetes duration on the muscle weakness was investigated by Andersen et al. they observed that the patients with long-term T2DM more than 20 years had lower peak isokinetic torque of knee EXT and FLEX than age, sex and BMI matched control subjects which was related to the severity of neuropathy while the patients with diabetes duration less than 10 years had only the significant weakness in the FLEX peak torque [2,3]. The 485 T2DM in comparison with 2133 health subjects showed that the maximal power of knee extensor of diabetic men was lower than the age and sex matched health subjects, the quality of muscles in both gender was decreased in the patients which this weakness was correlated with HbA1c and diabetes duration [7,27]. The decrease of physical activity also is the important factor to increase muscle weakness in the T2DM patients [6,28,29]. Based on the previous studies, the current study has tried to control the effect of gender, physical activity, BMI, age and peripheral vascular impairment in the leg by case matching and use the ANCOVA test to report reliable outcomes as possible.

There are two probable reasons for weakness in the diabetes, muscle and neuromuscular impairment. Sarcopenia, change of fiber distribution, change of GLUT4 density, mitochondria and intramuscular fat and metabolic inflexibility are mentioned as muscular factors to cause weakness. Although high percent of these changes are not dependent to diabetes because it can be seen in the obesity health subjects and may be related only to insulin resistance [8,12,30,31] but when the diabetic patients were compared with age, sex and BMI matched health subjects, the more muscle weakness in the diabetic group was observed yet [2,3,7,27]. The second factor is neuromuscular impairment in the diabetes. Andreassen et al. found that a down regulation of neurotropic factors activity such as neurotrophin 3 (NT-3) and neurotrophin 4 (NT-4) released from end of motor and sympathetic axons to periphery in the T2DM and the amount of downregulation was significantly more in patients with than without neuropathy and correlated to gastrocnemius strength [32]. The correlation between the lower limb strength and the intensity of neuropathy has been confirmed in the previous studies [2,3] by which the second theory is reinforced.

In the current study the isometric MPT of knee flexor was not enough sensitive to show significant difference because in the 75 degree of knee flexion, the hamstring muscle is not in the optimal length to exhibit maximal effort [33]. The patients were not also classified based on a standardized clinical neurological examination to detect the intensity of neuropathy but each patient without any intermittent claudication, foot ulcers and abnormal ABI was included since these symptoms occur in the advance stage of neuropathy [34].

### Muscle endurance

The gender did not significantly effect on endurance indices unless EXT FI in the women who showed more fatigue than men during isokinetic protocol. The diabetes did not develop influence significantly on the endurance indices in the extensor versus flexor muscles. FLEXBA and RATIOBA indicated the HC subjects could sustain the isometric MPT of knee flexor after isokinetic protocol better than both diabetic groups. The surprising, FLEX FI and slope, the decline of force during 40 repetitions, was less in theT2MD > 10 Y group than T2MD < 10 Y and even HC group and the correlation analysis confirmed that the increase of years of diabetes correlated with less negative flexion slope. the result of the current study is accordance with Andersen et al. which illustrated less decline in endurance index of flexor and extensor of knee and ankle during 30 repetitions with velocity of 180 degree/s in the 44 T2DM patients with more than 20 years duration of diabetes in comparison with 44 age, BMI and physical activity matched health subjects. There was no significant correlation between endurance index and HbA1C, blood glucose and the intensity of neuropathy [1]. The study of IJzerman et al. in which the duration of diabetes in the patients groups has not been reported, showed the T2DM without neuropathy had been fatigued more than HC only in the knee flexor during 20 repetitions isokinetic with velocity of 120 degree/s [6]. It seems that the different results of two mentioned study may be related to chronicity of diabetes, since in the current study that patients were classified based on diabetes duration, both results were observed. in the our study, some confounding factors in the endurance level in the diabetic patients such as physical activity and peripheral arterial disease [1] were control in order to achieve reliable outcomes. Different responses seen in the knee flexor and extensor may be related to the different distribution muscle fibers and test conditions applied in this study too [1,6]. The knee flexor and extensor muscles consist of approximately equal percentage of fast and slow muscle fibers but the percent of slow fibers is dominant in the quadriceps [35] and fast fibers are dominant in the hamstring [36,37]. It means the hamstring muscle is less resistance to fatigue but has more glycolytic capacity for anaerobic activity than quadriceps [11]. Therefore, in diabetes which muscle fibers shift to type 2 [13,14], the knee flexor is susceptible to show significant changes. However, less loss in flexor muscles effort during isokinetic protocol observed in this study may be related to the more available fuel of knee flexors i.e. glycolytic source to compare extensor muscles. On the other hand, regarding to the test condition, knee extensor opposed to both the load of moment arm and gravity while the knee flexor only resisted to moment arm so the extensor worked more and was susceptible to more force decline than flexor.

The measurement of isometric MPT as index to assess the muscle fatigue has not been done up to now. Accumulation of more metabolic substances after isokinetic protocol may be responsible for more fatigue in the knee flexor of the diabetic patients than HC group because all sequences of test were anaerobic and dominant source to apply energy was glycolic [38]. On the other hand, the level of lactate and pyruvate in the T2DM are higher than health subjects [39] and one of complication of metformin therapy is rise of lactate [40]. The type 2 muscle fibers in which produce greater Phosphocreatine and glycogen degradation during exercise than the type 1 [41] gradually increase in the diabetes [13,14]. Based on mentioned topics, we can conclude that the accumulations of metabolic substances such as ADP, PCr and lactate after isokinetic protocol in the muscles of diabetic patients greater than HC group then the force production was more inhibited in the patients. The unexpected result seen in this study was more isotonic resistance of theT2MD > 10 Y in comparison with T2MD < 10 Y group which may be explained as compensatory and adaptive mechanisms related to develop of diabetes. Several studies confirmed that in the T2DM the number of type 1 muscle fibers and density of GLUT4 of slow fibers, number of capillaries around muscle fiber and oxidative capacity of muscle are decreased and the number of type 2B muscle fibers and glycolic capacity of muscle are increased [13,14,17,30,42,43]. Oberbach et al indicated that the oxidative and glycolytic enzymes of the all types of muscles fibers and the density of GLUT4 in the type 2 muscle fiber were increased in the T2DM but the duration of diabetes of patients was not clear in the study [14]. On the other hand, the obese health subjects with high waist-to-hip ratio showed the same abnormalities like diabetic patients in muscle morphology, namely, a low percentage of type 1 fibers, elevated type 2 fibers (particularly type 2B) with a low capillary density. But the density of GLUT4 in both muscle fiber types was reduced more than the control and diabetic groups. It seems the changes in the obese subjects were only related to muscle insulin resistance because of high fat content [13,17,30]. Changes of the distribution of muscle fibers, the amount of oxidative and glycolytic enzymes can be affected on the ways to produce energy for muscle actions and the level of strength and endurance [11,43,44]. Unfortunately, most of the mentioned studies did not report the duration of diabetes in the patients whereas the duration of diabetes is main risk factor to progress of vascular and neural deficits [45,46] and sarcopenia [12,47] then it is expected that the muscle impairment is to be correlated with diabetes worsening [4,22,48]. It seems that the increase of hyperglycemia and insulin resistance result in shifting the muscle fibers to type 2 which are able to adapt to changes affected by diabetes [11,16,17,31,43]. Therefore, to compensate loss of type 1 muscle fiber function and preserve maximal performance, the oxidative and glycolytic capacity of the type 2 muscle fibers gradually will be raised [14]. Mitochondrial function studies in the progressive aerobic exercise demonstrated that the T2DM with history of disease around 6 years exhibited different mitochondria activity in comparison with patients that had more than 12 years history of disease. The short-term diabetic group had longer phosphocreatine recovery half time (PCr half-time) than BMI-matched health group whereas IMCL (intramyocellular lipid concentration) content was similar while the long-term diabetic group had higher IMCL content with no significant difference in the parameters for mitochondrial function, i.e. PCr and ADP recovery time [49,50]. It suggested that compensatory mechanism related to long-term diabetes in order to increase the muscle performance might be occurred. As our results showed the patients with long-term more than 10 years diabetes had more ability to maintain muscle force during intensive short repetitive contractions in the postural muscles such as knee flexor than the T2MD < 10 Y group and was closer to HC group. Our findings are only attributable to the anaerobic condition because the duration of isokinetic protocol was around 50 seconds in which the dominant source of energy is glycolytic system and oxidative circles were not involved completely [38,41].

## Conclusions

It is concluded that patients with long-term diabetes more than 10 years had more decline in the muscle strength but less decline in the knee flexor work during intensive short isokinetic protocol than sex, BMI, ABI and PAI-matched HC and T2DM < 10 Y groups. It is assumed that generally the muscular and neuromuscular deficits increase by increase years of disease [4,22] according to increase of hyperglycemia and insulin resistance but the maturation of muscle fibers to type 2 has been completed [14] and glycolic source has been raised. Then the capacity of muscle to supply energy for short activity is provided even more than HC group while in early stages of diabetes this compensation has not been completed. The isometric and isotonic of strength and endurance indices were lower than age, sex, BMI, ABI and PAI-matched HC group.

## Abbreviations

T2DM: Type 2 diabetes mellitus; MPT: Maximal peak torque; FPG: Fasting plasma glucose; HbA1C: Haemoglubin A1C; ABI: Ankle brachial index; PAI: Physical activity index; BMI: Body mass index; MVC: Maximal voluntary contraction; FI: Fatigue index; EXT: Extension; FLEX: Flexion; HC: Health control; ADP: Adenosine diphosphate; PCr: Phosphocreatine; GLUT4: Glucose transporter type 4.

## Competing interests

The authors declare that they have no competing interests.

## Authors’ contributions

BH: Conception and design of study, Collection and possession of data, Statistical analysis and Drafted the initial manuscript, FB: supervised the study design and data analysis. MRMT: coordination for patients testing and revision of the manuscript. All authors read and approved the final manuscript.
